# The Efficiency of Plant Defense: Aphid Pest Pressure Does Not Alter Production of Food Rewards by Okra Plants in Ant Presence

**DOI:** 10.3389/fpls.2021.627570

**Published:** 2021-03-15

**Authors:** Akanksha Singh, Veronika E. Mayer, Sharon E. Zytynska, Benjamin Hesse, Wolfgang W. Weisser

**Affiliations:** ^1^Chair for Terrestrial Ecology, Department of Ecology and Ecosystem Management, Technische Universität München, Freising, Germany; ^2^Agricultural Ecology Group, Department of Environmental Systems Science, ETH Zurich, Zurich, Switzerland; ^3^Department of Botany and Biodiversity Research, Division of Structural and Functional Botany, University of Vienna, Wien, Austria; ^4^Department of Evolution, Ecology and Behavior, Institute of Infection, Veterinary and Ecological Sciences, University of Liverpool, Liverpool, United Kingdom; ^5^Land Surface-Atmosphere Interactions, AG Ecophysiology of Plants, Department for Ecology and Ecosystem Management, School of Life Sciences Weihenstephan, Technische Universität München, Freising, Germany

**Keywords:** pearl bodies, ant preference, aphid honeydew, plant defense, pests, plant performance

## Abstract

Pearl bodies are produced by some plant species as food reward for ants and in exchange, ants defend these plants against insect pests. Sap-sucking pests such as aphids also excrete honeydew as food reward for ants, leading to potential conflict where ants could preferentially defend either the plant or the aphid. How pest insects might influence plant pearl body production, is yet to be investigated. Okra is a widely consumed vegetable worldwide and is attacked by the ant-tended cotton aphid. The plants produce pearl bodies, which are predominantly found on the underside of the leaves and formed from epidermal cells. We conducted a set of field and greenhouse experiments to explore plant-aphid-ant interactions, their influence on pearl body production and resulting performance of okra plants, across okra varieties. We found that ants of *Pheidole* genus, which are dominant in okra fields, preferred pearl bodies over aphid honeydew; although, their highest abundance was recorded in presence of both these food rewards, and on one okra variety. Removal of pearl bodies from the plants increased their production; however, plant growth and chlorophyll content were negatively associated with pearl body replenishment. Potentially to mitigate this resource cost, plants developed such a novel defense response because we found that aphid presence reduced pearl body production, but only when there were no ants. Finally, aphids negatively affected plant performance, but only at very high densities. As aphids also attract ants, plants may tolerate their presence at low densities to attract higher ant abundances. Our study highlights that plants can adapt their defense strategies in pest presence for efficient resource use. We suggest that understanding pearl body associated interactions in crop plants can assist in using such traits for pest management.

## Introduction

Plants have evolved numerous defense traits against herbivorous pests that reduce pest growth (Walling, 2000; Mithöfer and Boland, 2012). One defense trait is the production of food rewards that plants produce as a nutrient resource to attract other species (Heil and McKey, 2003; Heil, 2015). Ants in particular have been shown to protect plants from herbivores in exchange for food rewards (Rosumek et al., 2009; Mayer et al., 2014). However, ants can also form mutualistic associations with the herbivores; e.g., ants tend aphids for their honeydew reward, and in return protect aphids from their natural enemies (Buckley, 1987; Völkl et al., 1990; Kaplan and Eubanks, 2005). Benefits and costs for plants through such mutualistic ant-aphid interactions is unclear as plants can benefit indirectly from these interactions (Styrsky and Eubanks, 2007; Singh et al., 2016). Furthermore, in most ant-plant or ant-aphid interactions, protection by ants is context dependent. Hence, in the presence of ant-tended aphids, plants may develop different strategies to promote plant defense.

Pearl bodies are one of the food rewards produced by plants. These are found on plant surfaces with lustrous pearl-like appearance and are easily detached from the plants by ants (O’Dowd, 1982). Pearl bodies of some plant species are known to be a high-energy food source for ants due to their large content of lipids, amino acids and carbohydrates (O’Dowd, 1982; Heil et al., 1998; Fischer et al., 2002; Webber et al., 2007). In several specialized ant-plant systems, pearl body production is increased when pearl bodies are harvested by ants (Risch and Rickson, 1981) and also when they are removed artificially (Folgarait et al., 1994). This implies that production of pearl bodies is costly for plants (Mayer et al., 2014). In fact, plants have been found to allocate around 10% of their aboveground biomass to food body production (Heil et al., 1997; Fischer et al., 2002). Increases in plant fitness via ant protection against herbivores possibly balances out the negative cost borne by the plant (Heil, 2008; Rosumek et al., 2009).

The majority of studies investigating pearl body production have so far been conducted on specialized obligate ant-plant associations such as those found in *Cecropia*, *Macaranga* or *Piper* plants (Risch and Rickson, 1981; Fiala and Maschwitz, 1992; Folgarait et al., 1994; Fischer et al., 2002). In such associations, ants always defend plants against herbivores and, plants invest in pearl body production, irrespective of the fitness cost. Pearl bodies have rarely been investigated in facultative (context-dependent) ant-plant associations (Becerra and Venable, 1989; Rico-Gray et al., 2008; Paiva et al., 2009). When ant-plant associations are facultative, it can lead to conditional mutualism where the association may be mutualistic in one habitat, or time period, and antagonistic (or neutral) in another (Becerra and Venable, 1989; Rico-Gray et al., 2008). Furthermore, most ant associations with herbivores such as aphids are also facultative and vary with a number of factors (Stadler et al., 2001; Stadler and Dixon, 2005). Hence, when a facultative ant-associated aphid feeds on a facultative ant-associated plant, competition may arise between plants and aphids for ant protection, influencing plant production of pearl bodies. To our knowledge, prior studies on facultative ant-plant/ant-aphid systems have only focused on the role of ants and have ignored the role of herbivores such as aphids in pearl body production. Effects of ecological interactions are better understood by moving beyond pairwise interactions of adjacent trophic levels, e.g., ant-plant, and including interactions that occur across multiple trophic levels, e.g., ant-aphid-plant (Abdala-Roberts et al., 2019).

Herbivores influence plant physiological processes, for example by reducing the water potential (Cabrera et al., 1995) or photosynthetic efficiency (Ni et al., 2002; Macedo et al., 2003). Hence, herbivores may affect plant investment in food reward production and consequently the ant-plant association. This has mainly been investigated for another plant food reward, extrafloral nectar (EFN). For example, a study on *Catalpa bignioides* demonstrated total EFN volume and the secretion of sugars in EFN to increase two to threefold after herbivore attack; this in turn significantly increased ant presence on the plant and the consequent protection against herbivores (Ness, 2003). Plant food rewards can also differ across plant varieties (Wooley et al., 2007) and this can cascade to influence ant-plant-herbivore association. Such effects were shown in cotton plants (Rudgers, 2004), where variation in EFN production influenced the abundance of nectar-feeding ants and the consequent reduction of cotton herbivores. Effects of herbivores on food reward production and variation in this response across plant varieties have not been investigated for pearl bodies. Such information can be crucial for agricultural systems to select for plant varieties with enhanced plant defense potential.

Okra (*Abelmoschus esculentus* Moench, family: Malvaceae, order: Malvales), an economically important vegetable crop, produces pearl bodies on its stem and leaf surfaces. An annual survey conducted by the Singh (2011) found the facultative ant-tended cotton aphid (*Aphis gossypii* Glover, family: Aphididae, order: Hemiptera) to be the dominant pest on okra; ants of genus *Pheidole* (family: Formicidae, order: Hymenoptera) to be present on okra plants in 75% of the surveyed okra farms (IITA Annual Survey Report, Unpublished) and; farmers to grow multiple okra varieties.

In a previous field study, we found that although ants fed on aphid honeydew, no ant species protected aphids from their natural enemies. Instead ant presence reduced herbivory by another pest, the leaf beetle (*Nisotra uniformis* Jacoby, family: Chrysomelidae, order: Coleoptera) (Singh et al., 2016). *Pheidole dea* Santchi ants were recorded to be the dominant ants on okra plants in our field study and in controlled greenhouse conditions these ants were even observed predating on the aphids; with some variation in this ant-aphid interaction across okra varieties (Singh et al., 2016). We also observed pearl bodies to be present on leaf and stem surfaces of okra plants.

We conducted field and controlled experiments to uncover the role of pearl bodies in ant-aphid interactions. Identifying plant varieties that produce high numbers of pearl bodies and are favorable to ants could be beneficial to control herbivore populations in okra fields. Therefore, in this study we have also focused on the effects of plant varieties in mediating these pearl body associated interactions. We investigated the following questions: (a) Does okra pearl body production vary across okra varieties? (b) Do ants prefer okra pearl bodies over aphid honeydew, and does this vary across okra varieties? (c) Does aphid presence and (artificial) pearl body removal affect okra pearl body replenishment, and does this vary across okra varieties? We have further explained okra pearl body morphology in the current study as it has not been described before. Finally, in a third controlled experiment we tested if high aphid densities or pearl body production were costly for okra plants by investigating their effect on photosynthetic efficiency and water potential of the plant. Here we used aphid densities at the higher end of what is expected in the field to challenge the plants since previous experiments found little impact on plant growth.

## Materials and Methods

### Study System

Okra (*Abelmoschus esculentus* Moench) is mostly grown in humid climates in sandy and clay loam soils and its optimum growing temperature is 24°C to − 30°C. The plants are annual erect herbs (2–4 m tall) with lobed and hairy leaves. It is a self-pollinating crop but insects, especially bees are attracted to the flowers and hence cross pollination occurs (Tripathi, 2011). In the different experiments, we used up to five varieties of Okra: *Clemson, Hire, Paysan* (marketed by Les Doigts Verts, France), *Kirikou* (obtained locally from Dschang town, Northwest Cameroon), and *Caffeier* (G.M.R. Sarl, Cameroon).

Okra plants are attacked by many pests including cotton aphids (*Aphis gossypii* Glover) and leaf beetles *(Nisotra uniformis* Jacoby*)* (Benchasri, 2012). Cotton aphids colonize more than 600 host plants across a wide geographic range and vector more than 50 plant viruses (Van Emden and Harrington, 2007). In tropical climates, this facultative ant-tended aphid undergoes mostly parthenogenetic (i.e., asexual) reproduction, leading to an exponential growth rate at optimal conditions.

*Pheidole* is one of the most diverse ant genera in the world. *Pheidole* ants are particularly dominant in the tropics, with ∼900 species known worldwide (Wilson, 2003; Economo et al., 2015). Most colonies can consist of multiple queens and *Pheidole* species are often dimorphic, which means that the workers are subdivided into relatively slender minors, and stronger, conspicuously large-headed majors (Wilson, 2003).

### Experiment 1: Ant Visitation to Plant Pearl Bodies and Aphids

The aim of this experiment was to test which plants would attract higher abundance of *Pheidole* ants, plants with pearl bodies or plants with aphid honeydew. We further tested if this would vary across different varieties of okra.

The experiment was conducted in 2014 within the research site of the International Institute of Tropical Agriculture (IITA) research station in Yaoundé, in Cameroon (West Africa). Our study consisted of cotton aphids on okra, ant species of *Pheidole* genus and, five okra varieties (*Clemson, Hire, Paysan, Kirikou*, and *Caffeier*). The aphids used in this experiment were obtained from IITA where they were reared on *Clemson* variety, inside entirely enclosed plastic-polypropylene insect cages (1,350 μm mesh opening, 30 × 30 × 30 cm dimensions, Megaview Science, Taiwan).

The seeds were soaked in water under complete darkness for 24 h. Then one seed per pot (10 cm deep, 16 cm diameter) was sown in sterilized soil (25% sand, 25% fowl manure and 50% field soil) and left to germinate. The pots were kept inside entirely enclosed cages to avoid settling of pest species. Plants used for this experiment were 5 weeks old from the date of sowing.

#### Experimental Design and Set-Up

We used a fully factorial randomized block design with 20 treatments including five okra varieties, two aphid treatments (presence and absence) and two pearl body (PB) treatments (PB kept and PB removed), i.e., 5 varieties × 2 (−PB/ + PB) × 2 (−Aphid/ + Aphid) = 20. Due to an insufficient number of *Caffeier* and *Clemson* plants, (−)Aphid(−)PB treatments for these varieties were repeated only 11 times and distributed within 11 blocks. We repeated each of our other 18 treatments 12 times and distributed them within 12 blocks with each of the blocks containing one repeat of each of these 18 treatments. Hence, we had 238 plants in total. We staggered the set-up and data collection due to time constraints (temporal blocking), which means data were collected across 2 days for every plant within a block, with blocks distributed over time (week 1: blocks 1–6, week 2: blocks 7–9, and week 3: 10–12). We collected data for all treatments every week.

**FIGURE 1:**
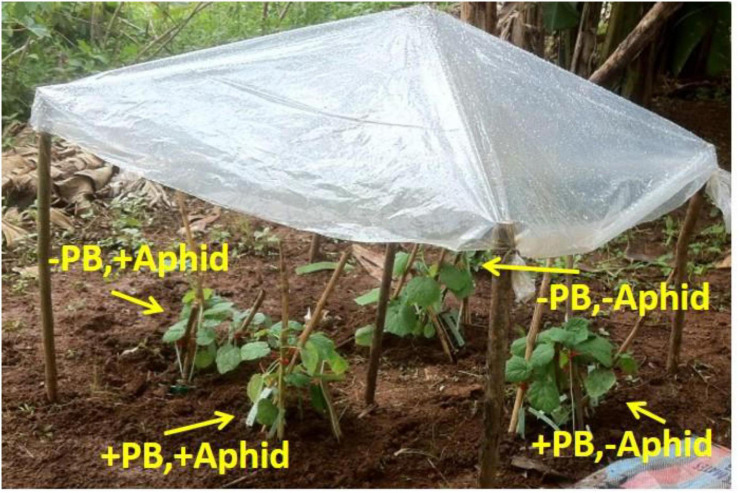
Experimental set up in the field to test preference of different ant species of aphid honeydew or okra pearl bodies. The image shows set up of one replicate. The plants are placed in four groups around the ant colony; each group includes all five plant varieties. Group 1: (–)Aphid (+)PB, Group 2: (–)Aphid (–)PB, Group 3: (+)Aphid (+)PB, Group 4: (+)Aphid (–)PB.

Two days before the experimental plants were placed outside for observation, *Pheidole* ant colonies were marked in the field site of IITA. Out of the ant colonies selected for observation, four colonies were of *Pheidole dea* Santschi, three of *Pheidole nigeriensis* Santschi, three of an unidentified *Pheidole* sp. (*Pheidole* 1) and two of another unidentified *Pheidole* sp. (*Pheidole* 2). Above each ant colony we built a water shelter using wooden sticks and a plastic cover of ∼120 × 100 cm (length × breadth) dimensions (Figure 1), to reduce the impact of heavy rain on the plants. All the vegetation around the ant colony was removed.

A day before the experimental plants were placed outside, we measured their height and leaf number. Pearl body numbers were also recorded but only on the plants (leaves and stem) with PB kept treatment; PBs were removed from the plants with PB removed treatment using a paint brush. Then 50 aphids of mixed instars were placed on the plants using a fine paint brush, to allow aphids time to settle on the plants. The next day we placed the experimental plants into the ground around the ant nest, to allow ants access to the plants. We buried the pots into the ground, until the level of the top of the pot and the ground was the same. Treatments were grouped around a single *Pheidole* species ant colony. Thus, each ant colony marked one block and all the treatments were present at each colony.

The treatments were grouped around an ant colony with respect to aphid and PB treatment, e.g., all varieties with aphid presence and PB removed were placed closer together as a group. Thus, we had four groups around each ant colony (aphid presence + PB kept, aphid presence + PB removed, aphid absence + PB kept and aphid absence + PB removed) and plant varieties were randomly placed within a group. Each of the groups (not plants) was placed at equal distance from the ant colonies and within a group all plants touched one another (Figure 1).

#### Data Collection

Plants were placed outside in the early morning of the first day. Each treatment group within a block was then observed for five minutes, repeated every 3 to 4 h for 2 days (during daylight hours); resulting in six total observations for each treatment group. We observed the number of ants and ant species on a plant and recorded separately if they were tending aphids or collecting pearl bodies. PBs were removed after each observation for PB removed plants in case new ones had been produced.

The average temperature during the experiment was 24.1 ± 0.2°C and average humidity was 89.4 ± 0.6%. Average rainfall was 9.9 ± 3.8 mm with natural 12:12 (light:dark) cycle.

### Experiment 2: Pearl Body Production Experiment and Pearl Body Morphology

The aim of this experiment was to investigate pearl body count variation among varieties as a function of aphid presence and pearl body removal (artificial removal to simulate ant collection), under controlled conditions. We also investigated the consequences for plant growth. We could not include one of the varieties (*Caffeier*) in this experiment as it failed to germinate in our experimental soil.

Experiments 2 and 3 were conducted in the greenhouse at the Dürnast experimental station of Technical University of Munich in Freising, Germany. Experiments 2 was conducted in February 2015 and experiment 3 in July 2016. During these experiments temperature of the greenhouse was 24/20°C (day/night) and additional lighting of 16:8 h (light:dark) was used. Considering artificial and natural sunlight, we can approximate that light intensity in experiment 2 ranged from 40 to 210 μmol/m^2^^∗^s and in experiment 3 from 210 to 1440 μmol/m^2^^∗^s. The cotton aphids used for both experiments belong to the ‘Darmstadt’ strain. These were reared on *Clemson* variety of Okra at temperatures of 20°C/18°C and 16:8 (light:dark) hours light cycle to maintain asexual reproduction, in plastic-polypropylene fine mesh insect cages.

Seeds from four okra varieties (*Clemson*, *Paysan*, *Kirikou* and *Hire*) were soaked in water under complete darkness for 24 h. Then one seed per pot (9 cm deep, 10 cm diameter) was sown in potting soil (Einheitserde profi substrat, Germany) and left to germinate inside the greenhouse. Plants used were 5 weeks old.

#### Experimental Design and Set-Up

We used a fully factorial randomized block design with 16 treatments including four okra varieties, two aphid treatments (presence and absence) and two pearl body treatments (PB kept and PB removed) i.e., 4 varieties × 2 (PB −/+) × 2 (Aphid −/+) = 16. Each of our treatments was repeated ten times and distributed within ten blocks with each of the blocks containing one replicate of each treatment, giving us 160 plants in total. We conducted observations on the first five blocks on the first day and the next five blocks on the second day.

The day before the initiation of the experiment, we measured plant height (distance between the base of the stem at soil level to apex of shoot), and numbered all the leaves of each plant, and counted PB number separately for leaves and stems. After this, PBs were removed from plants using a paint brush (in the PB removed treatment). Then, 50 aphids of mixed instar were placed on each plant for the aphid present treatment. Each plant was placed on top of a plastic pot on a water-filled tray, to prevent the escape of aphids. Plants were then left for 1 week and watered daily.

#### Data Collection

One week after setting up the blocks, final plant height and final PB number were recorded for all plants, separately for each leaf and stem. After collecting PBs, we measured leaf area separately for each leaf of a plant. Leaf area was measured using a LI-3100 C area meter (LI-COR, United States). Additionally, leaves of similar successional stages from each of the varieties were submerged in FAA (formaldehyde – acetic acid – 70% ethanol) solution and sealed in zip lock bags for leaf and pearl body structure analysis.

#### Okra Pearl Body Structure

For morphological pearl body investigation, two young and two mature leaves were randomly chosen from each okra variety and virtually divided into 4 sectors: basal part left and right from the main rib, apical part left, and right from the main rib. From each sector two pieces of 5 mm^2^ were cut out, critical point dried and the upper and lower surface investigated with a Jeol JSM IT300 scanning electron microscope (SEM) at 10 kV. For anatomical investigation, two parts per leaf sector were taken from *Clemson* and *Paysan*, soaked and embedded in a resin based on hydroxyethylmethacrylate (Kulzer Histo-Technique), cut in 2.5–3 μm cross-sections with a microtome, stained with Ruthenium Red/Astra Blue (both Sigma Aldrich) and investigated with an Olympus BX50 microscope.

### Experiment 3: Pearl Body Production and Plant Physiology Across High and Low Aphid Densities

This final experiment further investigated the effect of different aphid densities on pearl body production, relative plant growth, photosynthetic efficiency and on the water potential of the plants. We further tested if these effects varied across okra varieties. Plant and aphid preparation proceeded as in experiment 2. Pearl bodies were not removed.

#### Experimental Design and Set-Up

We used a fully factorial randomized design with 12 treatments including four okra varieties (*Clemson*, *Paysan*, *Kirikou*, and *Hire*) and three aphid treatments [one aphid absence and two aphid presence (low aphid density and high aphid density)]. Each of the treatments were repeated five times giving us 60 plants in total and the plants were placed on four separate tables. We placed the plants on top of plastic pots on a water-filled tray (to prevent escape of aphids). To avoid the movement of aphids between different aphid treatments, a table was divided into three sections and each section contained one aphid treatment. The plants were distributed randomly on the tables within the respective aphid treatments. We first measured the plant height and recorded total number of PBs on all plants (leaves and stem). Then, for plants with aphid presence we placed an aphid-infected leaf, close to the stem of the plant. We placed ∼1300 aphids on plants with low and ∼2600 aphids on plants with high aphid density treatment, respectively.

#### Data Collection

Eight days after setting up the experiment, data were recorded on final plant height and total final PB count on all plants. Leaf samples were collected from each plant (see details below) for the measurement of their water potential and photosynthetic efficiency (chlorophyll content and photosynthetic quantum yield).

#### Water Potential Measurement

Water potential is expressed in negative numbers and the highest water potential in plants is zero (Chavarria and dos Santos, 2012). One leaf from each plant was cut using a razor blade and immediately after this, the leaf was placed into a scholander pressure chamber (model 1505D, PMS Instruments, United States) to measure its water potential.

#### Photosynthetic Quantum Yield Measurement

Photosynthetic quantum yield is an indicator of photosynthetic efficiency (Baker, 2008). One leaf per plant was first dark adapted for 20 min using Leaf Clips DLC-8 (Walz, Germany). The yield was then measured using Photosynthetic Yield Analyser Mini-Pam (Walz, Germany) together with the software WinControl 2.08 (Walz, Germany).

#### Total Chlorophyll Content

Discs (0.68 diameter and 0.36^2^ cm area) were collected from five upper leaves of each plant and stored in complete darkness at 4°C until extraction. Cool pure methanol was used for the extraction process. Samples were measured for their chlorophyll content within 30 min after the extraction. The extinction (E) of the chlorophyll pigments A and B was measured at wavelengths 665.2 nm and 652 nm with an Uvikon 940 photometer (Roche Diagnostics International AG, Switzerland). Calculations were done as shown by Porra (2002) for chlorophyll A and B in μg/ml.

Chlorophyll=A16.29×E665.2-8.54×E652

Chlorophyll=B30.66×E652-13.58×E665.2

Further, chlorophyll content per unit area was calculated for each of the units mentioned above. Afterward we summed the per unit area values to calculate total leaf chlorophyll content per plant.

Totalchlorophyllcontent=(Chl+A/areaChl)B/area×

totalleafareaperplant.

### Data Analysis

The data for all three experiments was analyzed using R version 3.3.2 in RStudio version 0.98.978. For all the variables that we tested, we used Type I sum of squares; we first fitted a full model with all main effects and all interaction between the main explanatory variables. Final models were chosen on the basis of lowest AIC (for mixed effect models) and highest adjusted R square (for linear models) values.

Correlation values reported in the result section were obtained using Pearson product-moment correlation method. Data in the text is given as mean ± SE.

#### Experiment 1: Ant Visitation to Plant Pearl Bodies and Aphids

Five plants were removed from the analysis, three of them [*Hire* (+Aphid-PB), *Clemson* (−Aphid + PB), and *Paysan* (+Aphid-PB)] were damaged due to strong wind and two of them [*Hire* (+Aphid + PB) and *Clemson* (+Aphid-PB)] were partially eaten by an unidentified spiny caterpillar within 6 h of putting the plants in the field. Hence, we had a final sample size of 233 plants and 11–12 repeats for all our treatments. For initial PB count as a response variable we ran a linear model (lm) with normal distribution (best model fit), including plant variety, aphid treatment and PB treatment as main explanatory variables. To test the effect of our treatments on the response variable *Pheidole* ant numbers, we ran generalized linear mixed effect models (glmer) with quasipoisson distribution (overdispersed data) including plant variety, aphid treatment (presence and absence) and PB treatment (PB kept and PB removed) as main explanatory variables and block as a random effect. When a significant plant variety effect was observed, we also conducted *post hoc* analysis using Tukey HSD test to compare the effect across different plant varieties.

#### Experiment 2: Pearl Body Production Experiment and Pearl Body Morphology

Plant relative growth rate (plant RGR) was used to correct for plant height variation amongst varieties; it was calculated using the formula: [ln (Final plant height) – ln (Initial plant height)]. We also calculated the following variables:

(a) Total leaf replenishment (PB kept plants) = [final total leaf PB count – initial total leaf PB count]; Total leaf replenishment (PB removed plants) = [final total leaf PB count –0].
(b) Leaf PB replenishment/cm^2^ = (leaf PB replenishment/total leaf area).

For all our response variables mentioned below we included aphid treatment (presence/absence), PB treatment (kept and removed) and plant variety as main explanatory variables. For total PB replenishment we applied glmer with quasipoisson distribution. For the response varaibles initial pearl body count, leaf PB replenishment/cm^2^ and plant RGR we appled linear mixed effect (lmer) models. Additionally, for plant RGR we included total replenished PB count as a covariate.

#### Experiment 3: Pearl Body Production and Plant Physiology Across High and Low Aphid Densities

Total PB replenishment was calculated as final PB count – initial PB count. Pearl bodies were not removed in this experiment. Data was analyzed for the following response variables: water potential, total chlorophyll content, photosynthetic quantum yield, total PB replenishment, and plant relative growth rate (plant RGR). For all our response variables, we ran lmer models including aphid treatment and plant variety as main explanatory variables, and table as a random effect. When a significant aphid effect was observed, we also conducted *post hoc* analysis using Tukey HSD test to compare the effect across different aphid treatments. For the response variable total PB replenishment, we included all other response variables as covariates except plant growth.

## Results

### Overall Variation in Pearl Body Count and Density Across Okra Varieties

The initial number of pearl bodies (PB) on plants (leaves and stem) varied across okra varieties (*P* < 0.001; Supplementary Table 1); highest PB numbers were observed on *Caffeier* (200.6 ± 13.38; Experiment 1) and *Clemson* [564.2 ± 19.41 (Experiment 2); 1666.4 ± 29.3 (Experiment 3); Supplementary Table 2]. Despite being the tallest, *Paysan* plants had the fewest numbers of PBs in each experiment (Supplementary Table 2).

### Ant Visitation to Plant Pearl Bodies and Aphids (Experiment 1)

**FIGURE 2:**
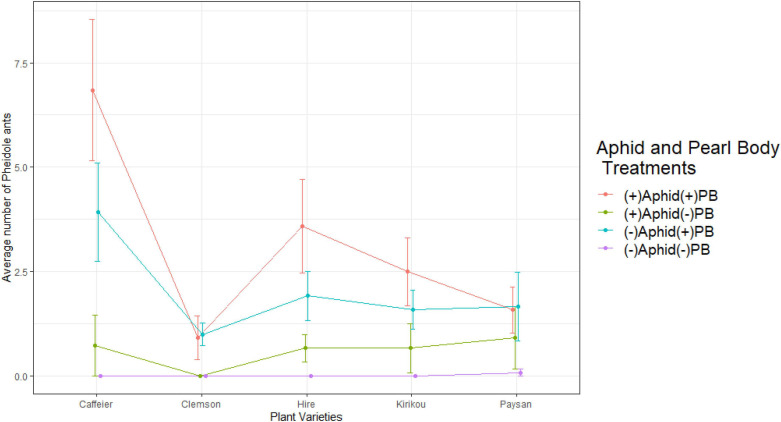
Variation in number of *Pheidole* ants across plant varieties, in different aphid and pearl body treatments. Pearl bodies were artificially removed for (–)PB plants. Error bars represent ± 1 SE.

*Pheidole* ants started visiting the plants within an hour of placing the plants around the ant colonies. Plant variety (*X*^2^ = 22.614, df = 4, *P* < 0.001) significantly affected the number of *Pheidole* ants and their highest total and average number was recorded on *Caffeier* variety of okra (Figure 2 and Supplementary Figures 1, 2). Plant variety effect on ant attraction was potentially mediated by *Clemson* variety as this was the only variety that significantly differed from all other varieties (*post hoc* pairwise analysis: *Clemson-Caffeier: t* = 4.637, *P* < 0.001; *Clemson-Hire: t* = 3.103, *P* = 0.016; *Clemson-Paysan: t* = 2.586, *P* = 0.072; *Clemson-Kirikou: t* = 2.511, *P* = 0.087).

We recorded a significant interaction between aphid treatment and PB treatment (*X*^2^ = 19.677, df = 1, *P* < 0.001), where *Pheidole* ant numbers were highest on plants in the presence of both food sources, however, when only aphids or PBs were present on a plant, higher, ant numbers were recorded in the presence of PBs alone. Ants were only observed once on plants with no aphids and no PBs (Figure 2). We also recorded a positive correlation between initial PB count and the number of *Pheidole* ants (*t* = 6.173, df = 238, *P* < 0.001, *r* =+0.371).

### Pearl Body Production Experiment and Pearl Body Morphology (Experiment 2)

We conducted a controlled experiment where we tested the effect of ant removal of PBs (PB removed vs PB kept plants) and of aphid feeding on the plants (aphid presence and absence) on PB replenishment and on plant growth. We analyzed data for both response variables, total PB replenishment (abundance after 7 days) and PB replenishment/cm^2^ (density). Since both showed the same patterns, we present only the total PB replenishment results.

Pearl body count increased on all plants, but plants replenished more PBs when these were removed from the plant than when these were not removed (PB kept plants replenished 37.8% and PB removed plants replenished 67.2% of their initial PB count; Supplementary Table 3). PB replenishment also significantly varied across plant varieties (*X*^2^ = 61.93, df = 3, *P* < 0.001); overall *Hire* variety replenished the most PBs (201 ± 18.08) and plants of *Paysan* variety the least (122 ± 11.61).

**FIGURE 3:**
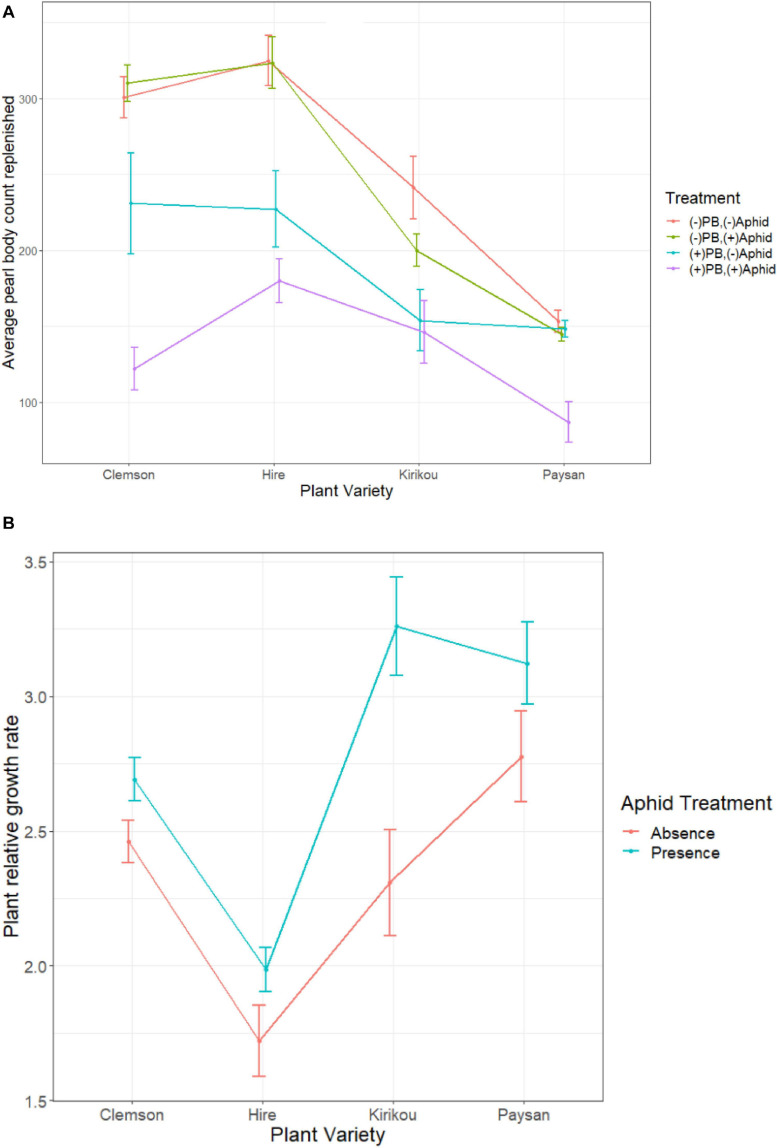
**(A)** Effect of aphid presence and absence on count of replenished pearl bodies, across different okra varieties in different pearl body treatments (PB Kept and PB removed). Pearl bodies were artificially removed at the beginning of the experiment only in PB removed plants. **(B)** Effect of aphid presence and absence on plant relative growth rate across different okra varieties. Error bars represent ± 1 SE.

We found a significant interaction between aphid and PB treatment on PB replenishment (*X*^2^ = 58.48, df = 3, *P* < 0.001); where aphids feeding on the plants (aphid presence) reduced PB replenishment but only when PB s were not removed. When PBs were removed (simulating ant collection), aphid presence did not reduce PB replenishment (Figure 3A). Although there was no significant 3-way interaction (*X*^2^ = 5.43, df = 3, *P* = 0.142), we observed an opposite effect for *Kirikou* variety, where aphid presence reduced replenishment only in plants where PBs were removed (Figure 3A).

Furthermore, aphids positively affected plant relative growth rate (RGR) and we recorded a significant two-way interaction between plant variety and aphid treatment on plant RGR (*F*_3_,_142_ = 2.88, *P* = 0.038); where aphid presence increased plant RGR for all varieties, but the highest increase was observed for *Kirikou* variety (Figure 3B). In a repeat of the experiment, we confirmed the potentially counterintuitive, but significant positive effect of aphids on plant RGR.

Pearl body treatment had no effect on plant RGR (*F*_1_,_142_ = 1.19, *P* = 0.277), however, there was a negative association between plant growth and number of PBs replenished (*t* = −3.84, df = 156, *P* < 0.001, *r* = −0.294). Plant RGR (*F*_3_,_142_ = 23.48, *P* < 0.001) varied across plant varieties but followed an opposite trend as of PB replenishment; *Paysan* plants grew the most (by 20.65 ± 0.80 cm) and *Hire* the least (by 12.95 ± 0.56 cm; Supplementary Table 4).

#### Okra Pearl Body Structure

**FIGURE 4:**
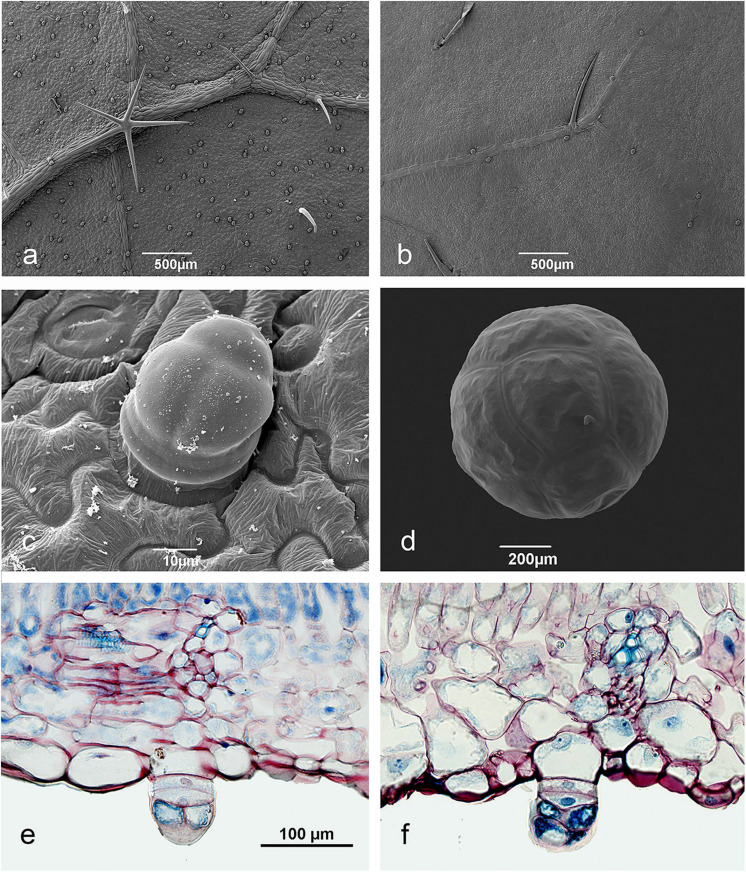
Pearl body structure in Okra varieties. **(a,c)** lower leaf surface of *Clemson* young leaf, **(b)** upper leaf surface *Clemson* young leaf, **(d)** mature pearl body from a *Hire* leaf, **(e,f)** longitudinal leaf sections of *Paysan* **(e)**, and *Clemson* **(f)** with pearl bodies developing on the lower leaf surface. The initial epidermis cell, the two stalk cells and the apical cells dividing anticlinally can be seen.

PBs occur on okra stems and both leaf surfaces, whereas, PB density is found to be much higher on the abaxial surface (Figure 4a). On the upper surface PBs are rare and occur only near the veins (Figure 4b). Okra PBs are multicellular and of epidermal origin, cells of the subepidermis are not involved (Figures 4e,f). Their elaboration is through periclinal cell divisions with subsequent anticlinal division of the two apical cells (Figures 4c,e,f). Two basal cells remain as the “stalk.” They are usually composed of eight cells surrounded by a very robust cuticle (Figure 4d). During their enlargement, the characteristic spherical shape is reached. Young leaves have plenty of initial stages of pearl bodies on their abaxial surface (Figure 4a) but on old leaves much lower numbers occur. This strongly suggests that PB replenishment rate is correlated with leaf age.

### Pearl Body Production and Plant Physiology Across High and Low Aphid Densities (Experiment 3)

**FIGURE 5:**
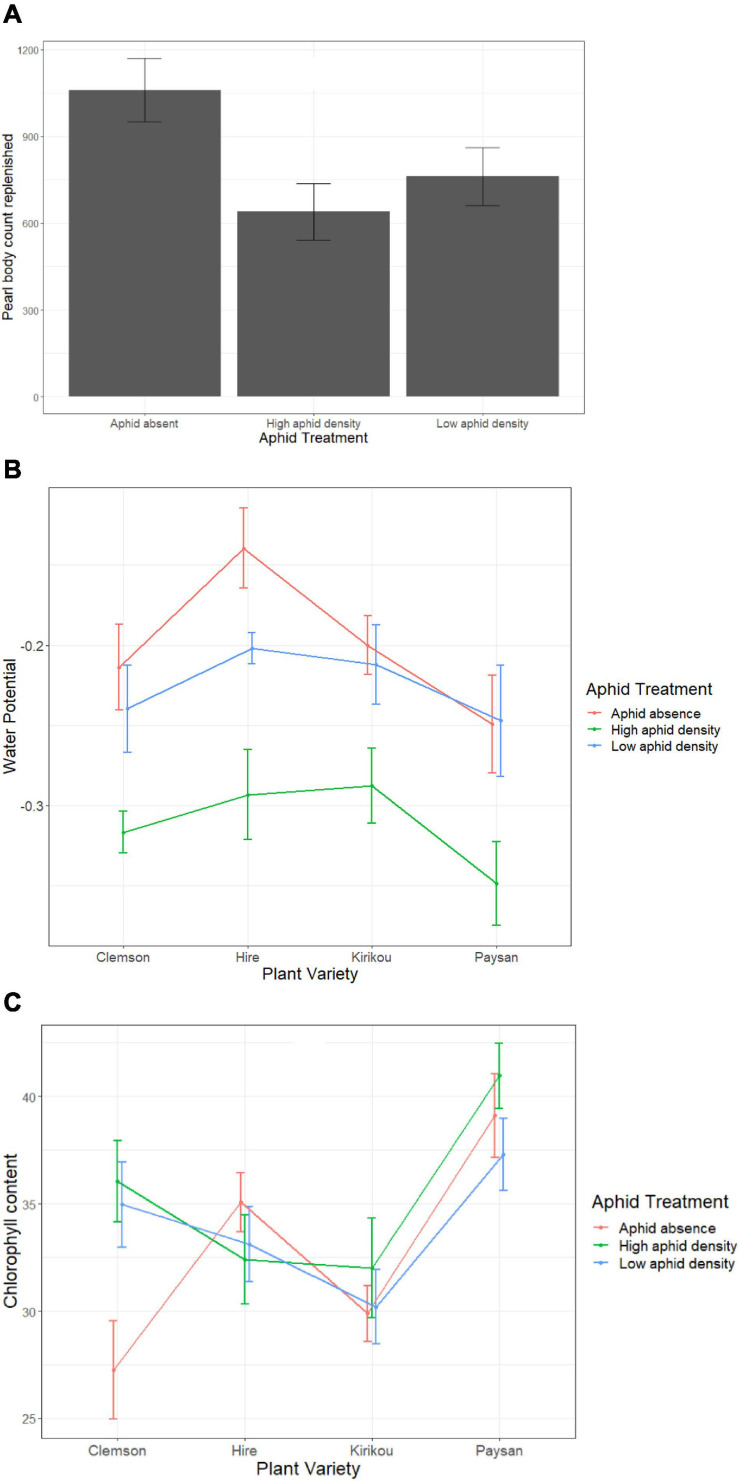
**(A)** Variation in pearl body replenishment at different aphid densities, **(B)** Variation in water potential across okra varieties at different aphid densities, **(C)** Variation in total chlorophyll content across okra varieties at different aphid densities. Error bars represent ± 1 SE.

Pearl bodies were not removed in this experiment. Similar to experiment 2 (for PB kept plants), the presence of aphids reduced pearl body production (*F*_2_,_51_ = 3.72, *P* = 0.031), but there was no difference between high and low aphid densities (*post hoc* pairwise analysis: *t* = 0.94, *P* = 0.619; Figure 5A). However, when water potential was included in our model as a covariate we found no significant aphid effect (*F*_2_,_49_ = 1.32, *P* = 0.276). Instead, we found a significant effect of water potential on pearl body production (*F*_1_,_49_ = 5.63, *P* = 0.022), where the number of pearl bodies were increased when plants had higher water potential. As aphid treatment negatively affected plant water potential (*F*_2_,_49_ = 30.77, *P* < 0.001; Figure 5B), this suggests that one of the mechanisms by which aphids reduce peal body production is may be driven by their effect on water potential. However, water potential reduced only at high aphid densities (aphid absence-high density: *t* = 7.468, *P* < 0.001, low-high density: *t* = 5.81, *P* < 0.001) and there was no significant variation between aphid absence and low aphid density (*t* = 1.66, *P* = 0.232; Figure 5B). Plant water potential was also affected by plant variety (*F*_3_,_49_ = 6.80, *P* = 0.023; Figure 5B).

Pearl body production was also affected by chlorophyll content (*F*_1_,_49_ = 4.14, *P* = 0.047) and increased with a decrease in chlorophyll content; suggesting that pearl body production can be resource intensive for okra plants.

We further found a marginally significant interaction between aphid treatment and plant variety on total chlorophyll content (*F*_6_,_45_ = 2.25, *P* = 0.056), where high and low aphid densities reduced chlorophyll content only in *Clemson* variety but had no effect on other okra varieties (Figure 5C). Overall, *Paysan* plants that produced the fewest PBs had the highest chlorophyll content (Figure 5C).

Even with the high aphid density in this experiment, we found aphids to have no negative effect on plant growth (*F*_2_,_49_ = 0.90, *P* = 0.413). Plant growth only varied across varieties (*F*_3_,_49_ = 5.26, *P* = 0.004) and was increased in plants with higher photosynthetic yield (*t* = 2.11, df = 58, *P* = 0.039, *r* = + 0.27), but this was not explained by differences in photosynthetic yield across plant varieties (*F*_3_,_50_ = 1.72, *P* = 0.174). Photosynthetic yield was also not affected by aphid treatment (*F*_2_,_50_ = 0.29, *P* = 0.219).

## Discussion

Our study shows that pearl body production and its variation across okra varieties can alter ant-plant interactions in the field. Ants of *Pheidole* genus were attracted in higher numbers to plants with pearl bodies than to plants with just aphids. Ant visitation also varied across okra varieties and was positively correlated with pearl body number. These results indicate that ants potentially preferred okra pearl bodies over aphid honeydew. This ant preference of pearl bodies may be one of the reasons why ants did not defend aphids against their natural predators in our previous study (Singh et al., 2016). Our results further showed that plants can adapt their production of food rewards in order to maximize, defense, and minimize negative effects on other physiological traits. We found a novel effect of aphid herbivores on pearl body production in our system, whereby aphids reduced pearl body production only when these remained on the plant (simulated ant absence). When pearl bodies were removed (simulated ant presence), their production increased, and aphids had no effect on their replenishment. Pearl body production was negatively correlated with plant growth and plant chlorophyll content. As pearl body production may be resource intensive for the plants (Heil et al., 1997; Fischer et al., 2002), plants potentially invest in higher pearl body production only when these are removed. The removal potentially suggests that ants have been recruited to the plant and can control the herbivore population (Offenberg, 2015; Thurman et al., 2019).

Effects of aphid infestation on pearl body production have not been investigated before. Studies on another food reward extrafloral nectar (EFN) have shown aphid infestation to increase or decrease EFN secretion. However, this has been found to be dependent on the identity of the aphid species or on the presence of other organisms (Jaber and Vidal, 2009; Yoshida et al., 2017). We also found context dependency in our study where aphid reduction of pearl bodies was dependent on pearl body removal. We suggest potential mechanisms for this variation in aphid effect. First, the reduction of pearl body replenishment in the presence of sap-sucking aphids (only when pearl bodies were not removed) may be due to nutrient loss (Guerrieri and Diglio, 2008). This was further evident in our third experiment where we found okra water potential to reduce at high aphid density, which indicates reduced potential energy in the system (McElrone et al., 2013). Studies on other pearl body bearing plants have suggested that good nutrient supply may be crucial for pearl body production (Folgarait and Davidson, 1995; Heil and Baldwin, 2002; Paiva et al., 2009). Second, pearl bodies contain more amounts of lipids and proteins (Heil et al., 1998; Fischer et al., 2002; Webber et al., 2007). Lipids and protein are a higher energy food source (Wäckers et al., 2001; Fischer et al., 2002) and potentially their production is costly for okra plants. Hence, to utilize their resources efficiently plants invest less in pearl body production in aphid presence when pearl bodies are not removed (signals ants’ absence). Finally, no aphid effect on pearl body replenishment when pearl bodies were removed may have occurred because pearl body removal can signal ant arrival. Food body production has been found to be inducible by ants (Risch and Rickson, 1981) and by their mechanical removal (Folgarait et al., 1994). Earlier studies have shown negative effects of pearl body production on aboveground plant biomass (Heil et al., 1997) and on plant growth (Frederickson et al., 2012). We found similar negative effects in our study, with both plant growth (height) and plant chlorophyll content negatively associated with pearl body replenishment. Additionally, *Paysan* variety plants, which produced the least pearl bodies were the tallest and had the highest chlorophyll content. Studies on EFN have also shown that ant presence can influence resource allocation by plants. For example, in one study in ant absence, plants reduced resource allocation to formation of extrafloral nectaries (Rutter and Rausher, 2004); whereas, in another study also in ant absence, plants increased production of direct defense compounds (Yamawo et al., 2015). Hence, when pearl bodies are not removed, okra plants probably do not use resources for their production because they are already present to attract ants. However, when pearl bodies are removed, plants potentially start to allocate more resources to pearl body production to continue to attract ants.

Overall, in our study, we found that aphids did not have negative effects on okra plant performance at low density. Instead, aphid presence increased plant growth. Plants are known to have various responses to herbivory such as overcompensation, where plant fitness is actually increased following moderate herbivore damage, and is higher than for non-attacked plants (Agrawal, 2000). There are several suggested mechanisms for such increased fitness, including the resource allocation mechanism (Trumble et al., 1993; Tiffin, 2000). This states that the distribution of available resources to a new site occurs at the expense of other metabolic centers (Trumble et al., 1993). As the cotton aphid mostly feeds on leaves, it may reduce leaf tissue resources, which may further cause utilization of stored plant resources for plant growth. Alternatively, as pearl body production reduced in aphid presence (only when pearl bodies were not removed), this potentially led the plant to distribute more resources to its growth. Although we recorded no overall effect of pearl body treatment on plant growth, we did record a negative association between number of replenished pearl bodies and plant growth. This was potentially driven by variation across varieties in plant growth and pearl body production. For example, the aphid effect on plant growth varied across okra varieties with *Kirikou* plants growing the most in aphid presence. This was also the only variety where aphid presence reduced pearl body replenishment when pearl bodies were removed (although no significant interaction was observed).

For all our variables (i.e., water potential, plant growth, and pearl body replenishment), we recorded significant variation across plant varieties. Crop varieties are bred for different traits (often for yield, fruit quality, or disease resistance) and it is not surprising that they would vary across such physiological traits. Aphids also significantly affected most of these variables and their effects were consistent across varieties. However, for plant growth and chlorophyl content, there was a significant aphid effect across varieties. Many studies have found plant-aphid interactions to be mediated by crop varieties or plant geno/biotypes (reviewed by Zytynska and Weisser, 2016). In our study, specifically, aphids increased plant chlorophyll, but only for the *Clemson* variety of okra. Generally, aphids are expected to reduce plant chlorophyll (Golawska et al., 2010) and thus this result is unexpected. Our controlled experiments ran for 1 week, it is possible that with continuous aphid pressure we would see a negative aphid effect on chlorophyll content of *Clemson* variety. We suggest that deeper investigation is needed of the mechanisms underlying these plant-aphid-ant interactions and to specifically test how these will ultimately relate to okra plant yield across different varieties.

In the experiment with high aphid density (∼2600 aphids/plant), okra plants suffered from a reduced water potential, which overtime will likely affect plant fitness (e.g., fruit production). In the absence of natural enemies, aphids can reach such high densities on okra plants. In okra fields in Cameroon however, aphid numbers usually do not reach such high densities when natural enemies are present (IITA Annual Survey Report, Unpublished). Hence, okra plants produce pearl bodies and may even tolerate aphid presence to attract ants and to reduce pressure from other more damaging herbivores.

Multiple pests attack plants and several studies suggest that plants may actually benefit from interactions between ants and honeydew producing insects due to ant reduction of other pests (Styrsky and Eubanks, 2007; Rosumek et al., 2009). Indeed, in our previous study, we found that ant attraction to the plant by aphids consequently resulted in reduction of another herbivorous pest, the leaf beetle. Furthermore, we also found that it was leaf beetles and not aphids that negatively affected okra yield (Singh et al., 2016). In our field study, highest ant numbers were recorded on plants where both food sources were present (aphid honeydew and pearl bodies) followed by plants with pearl bodies alone and then by plants with aphids alone. This suggests that although ants visit plants with maximal food rewards, they show a preference for pearl bodies over aphid honeydew. As mentioned above, honeydew, and pearl bodies have different chemical composition and the preference of ants to sugars or amino acids can be ant species-specific (Blüthgen et al., 2004). We also recorded higher numbers of these ants on *Caffeier* variety of okra, which potentially occurred due to the highest initial pearl body count on this variety. *Pheidole* ants are dominant ants in okra fields in Cameroon, and the attraction of *Pheidole* ants to pearl bodies and to particular varieties could be beneficial for development of pest control measures, as these ants provide no protection to the aphids (Singh et al., 2016). Through investigation of okra pearl body morphology, we found pearl body replenishment to be related with leaf age and to be higher on younger leaves. This further suggests the importance of pearl bodies as defense source for plants (McCall and Fordyce, 2010).

Our study explored ant-aphid-plant interactions involved in an inducible defense. We highlight how herbivores can induce multiple responses in a plant and may not reduce plant fitness at a density corresponding to field infestations (in the presence of natural enemies). Our study further shows that when plants produce a resource-intensive food reward such as pearl bodies, they optimize its production only when useful (i.e., in ant presence). This means that plants may tolerate the presence of a less damaging herbivore and limit pearl body production when ants are absent. Pearl bodies are prevalent in many plant families but are not utilized for developing pest control strategies. This may be because their associated interactions are context dependent, and thus assumed difficult to predict (Jones et al., 2017). We suggest that exploring interactions associated with food rewards in agricultural crops can provide missing insights for development of pest control measures.

## Data Availability Statement

The raw data supporting the conclusions of this article will be made available by the authors, without undue reservation.

## Author Contributions

AS, SZ, and WW designed the experiments. AS and BH collected the data. VM analyzed the pearl body structure. AS and SZ analyzed the data. AS and VM wrote the first draft of the manuscript, which was commented by SZ, WW, and BH. All authors read and approved the final version of the manuscript.

## Conflict of Interest

The authors declare that the research was conducted in the absence of any commercial or financial relationships that could be construed as a potential conflict of interest.
