# Supplementary material for: The Efficiency of Plant Defense: Aphid Pest Pressure Does Not Alter Production of Food Rewards by Okra Plants in Ant Presence
Source: Front Plant Sci. 2021 Mar 15;12:627570. doi: 10.3389/fpls.2021.627570 (PMC8005652; doi:10.3389/fpls.2021.627570)
Supplement: Supplementary file 1 [file Data_Sheet_1.docx]

# Supplementary material

# The efficiency of plant defense: Aphid pest pressure does not alter production of food rewards by okra plants in ant presence

*Akanksha Singh*^1,2^, Veronika E. Mayer^3^, Sharon E. Zytynska^1,4^, Benjamin Hesse^5^ and Wolfgang W Weisser^1^*

*^1^Terrestrial Ecology Research Group, Department for Ecology and Ecosystem Management, School of Life Sciences Weihenstephan, Technische Universität München, Hans-Carl-von-Carlowitz-Platz 2, 85354 Freising, Germany*

*^2^ Agricultural Ecology Group, Department of Environmental Systems Science, ETH Zurich, Universitätstrasse 2, 8092 Zurich, Switzerland*

*^3^Department of Botany and Biodiversity Research, Division of Structural and Functional Botany, University of Vienna, Rennweg 14, A-1030 Wien, Austria*

*^4^ Department of Evolution, Ecology and Behaviour**, Institute of Infection, Veterinary and Ecological Sciences, University of Liverpool, Liverpool, UK*

*^5^Land Surface-Atmosphere Interactions, AG Ecophysiology of Plants, Department for Ecology and Ecosystem Management, School of Life Sciences Weihenstephan, Technische Universität München, Hans-Carl-von-Carlowitz-Platz 2, 85354 Freising, Germany*

**Correspondance:*

*email: akanksha.singh@usys.ethz.ch*

# Plant morphological variation across okra varieties

Okra varieties differed in their initial height, leaf number and pearl body count across all experiments (Table SI 1). Plants of *Caffeier* variety had the highest number of initial pearl bodies in expt. 1. In experiments 2 and 3, plants of *Clemson* variety had the highest and, plants of *Paysan* variety had the lowest numbers of initial and final pearl bodies (Table SI 2).

In the first experiment, initial pearl bodies were counted only for plants with ‘PB Kept treatment’, therefore, we cannot calculate results of PB treatment for this response variable. In the third experiment PBs were not removed from any plants.

### Table SI 1: Effect of different plant varieties, aphid treatment (presence/absence) and pearl body (keep/remove) on initial plant height, leaf number and initial pearl body count. Initial leaf number was not counted in the third experiment.

| **Explanatory**  **variables** | **Response Variables** | | |
| --- | --- | --- | --- |
|  | Initial plant height | Initial leaf number | Initial pearl body count |
| Experiment 1 |  |  |  |
| Plant Variety | F_4,233_=18.28, *P*<0.001 | F_4,233_=2.70, *P*=0.001 | F_4,115_=17.10, *P*<0.001 |
| Aphid Treatment | F_1,233_=1.23, P=0.267 | F_1,233_=0.04, P=0.867 | F_1,115_=0.02, P=0.874 |
| Pearl Body Treatment | F_1,233_=0.41, P=0.523 | F_1,233_=0.21, P=0.161 | NA |
| Experiment 2 |  |  |  |
| Plant Variety | F_3,152_=211.13, *P*<0.001 | F_3,152_=4.52, *P*<0.001 | F_3,152_=108.47, *P*<0.001 |
| Aphid Treatment | F_1,152_=0.36, *P*=0.549 | F_1,152_=0.28, *P*=0.096 | F_1,152_=2.15, *P*=0.144 |
| Pearl Body Treatment | F_1,152_=0.18, *P*=0.674 | F_1,152_=0.17, *P*=0.11 | F_1,152_=2.37, *P*=0.126 |
| Experiment 3 |  |  |  |
| Plant Variety | F_3,51_=61.92, *P*<0.001 | NA | F_3,51_=33.77, *P*<0.001 |
| Aphid Treatment | F_2,51_=2.54, *P*=0.056 | NA | F_2,152_=0.06, *P*=0.528 |

Initial leaf number and initial pearl body count were not affected by aphid or pearl body treatments in any of the experiments (Table SI 1). Initial plant height was not affected by aphid or pearl body treatment in experiment 1 and 2. However, in experiment 3 we did record a marginally significant effect of aphid treatment on initial plant height. Plants with aphid absence were on an average 2.9±0.42 cms shorter than plants with low or high aphid densities (Figure S1 1). In our main manuscript we present results of aphid treatment on plant growth and not on final plant height. Therefore, this aphid effect on initial plant height does not influence the results that we present for experiment 3.

### Table SI 2: Initial pearl body count and final pearl body count across okra varieties

|  | Initial pearl body count | |  | Final pearl body count | | |
| --- | --- | --- | --- | --- | --- | --- |
|  | *Expt. 1* | *Expt. 2* | *Expt. 3* | *Expt. 1* | *Expt. 2* | *Expt. 3* |
| *Clemson* | 112.3±9.81 | 564.2±19.41 | 1666.4±29.3 | - | 751.2±37.38 | 2472.5±27.4 |
| *Hire* | 137.7±13.25 | 540.1±11.75 | 1449.7±14.5 | - | 741.0±24.40 | 2575.8±17.3 |
| *Kirikou* | 152.1±12.28 | 437.5±28.94 | 1549.3±16.0 | - | 652.3±41.87 | 2396.2±37.4 |
| *Paysan* | 58.2±5.92 | 223.3±7.83 | 714.0±10.6 | - | 345.3±15.38 | 1217.6±28.2 |
| *Caffeier* | 200.6±13.38 | - |  | - | - |  |

**Table SI 3: Number of replenished pear bodies in different pearl body treatments, across okra plant varieties**

| **Plant variety** | **Replenished PB count (PB Kept plants)** | **Replenished PB count (PB Removed plants)** |
| --- | --- | --- |
| *Clemson* | 176.2±6.54 | 305.6±2.98 |
| *Hire* | 204.6±4.92 | 324.1±3.76 |
| *Kirikou* | 150.1±4.62 | 221.3±3.91 |
| *Paysan* | 117.9±2.85 | 149.4±1.42 |

Plants of *Paysan* variety were found to be the tallest and of *Kirikou* the shortest in all experiments (Table SI 4).

**Table SI 4: Initial and final plant height variation across okra varieties**

|  | Initial plant height (cms) | | | Final Plant height (cms) | | |
| --- | --- | --- | --- | --- | --- | --- |
|  | *Expt. 1* | *Expt. 2* | *Expt. 3* | *Expt. 1* | *Expt. 2* | *Expt. 3* |
| *Clemson* | 21.1±0.77 | 41.9±0.45 | 40.7±0.19 | - | 60.0±0.39 | 55.6±0.19 |
| *Hire* | 16.3±0.76 | 31.5±0.48 | 31.6±0.14 | - | 44.4±0.68 | 43.0±0.16 |
| *Kirikou* | 16.6±0.57 | 25.6±0.87 | 30.7±0.29 | - | 45.1±1.41 | 42.6±0.48 |
| *Paysan* | 23.9±0.68 | 44.6±0.51 | 48.5±0.21 | - | 65.2±0.83 | 63.9±0.34 |
| *Caffeier* | 18.9±0.90 | - |  | - | - |  |

There was only a small difference (<1 leaf) between varieties in their leaf numbers except for *Clemson* and *Hire* which had the lowest leaf number in experiment 1 and 2 respectively (Table SI 5).

### Table SI 5: Initial leaf number in experiment 2 and 1

| **Plant variety** | **Initial leaf number**  **(expt.2)** | **Initial leaf number (expt.1)** |
| --- | --- | --- |
| *Clemson* | 6.2±0.13 | 3.8±0.11 |
| *Hire* | 5.9±0.12 | 4.4±0.11 |
| *Kirikou* | 6.8±0.11 | 4.0±0.13 |
| *Paysan* | 7.1±0.09 | 4.2±0.10 |
| *Caffeier* |  | 4.4±0.11 |


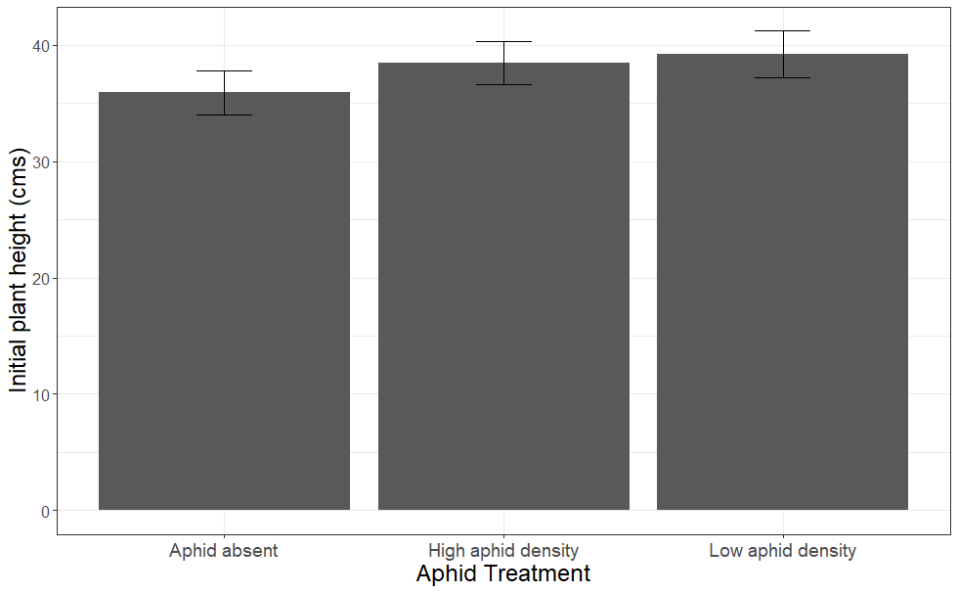


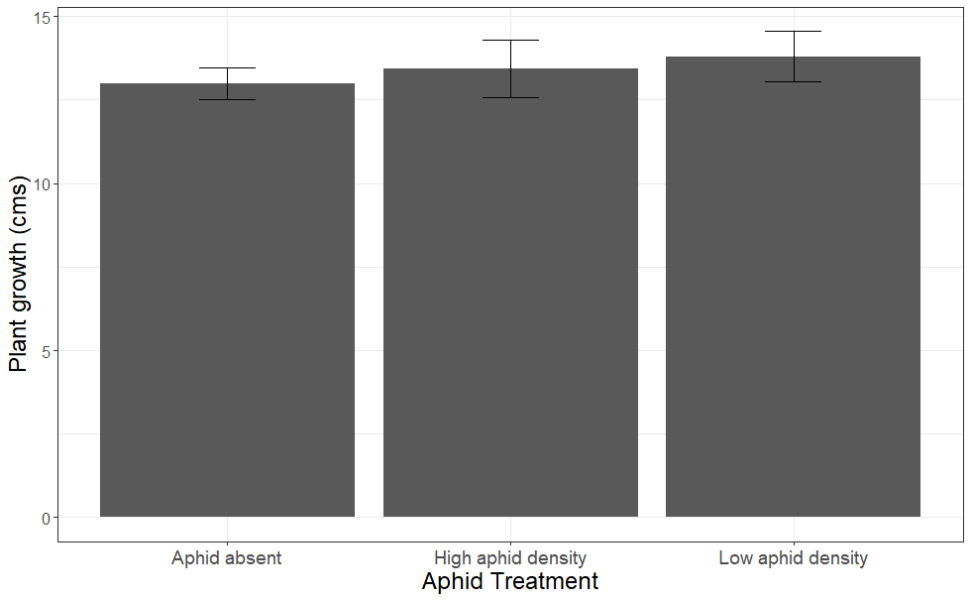


Figure SI 1: Initial plant height and plant growth variation across different aphid treatment in experiment 3. Error bars represent ±SE


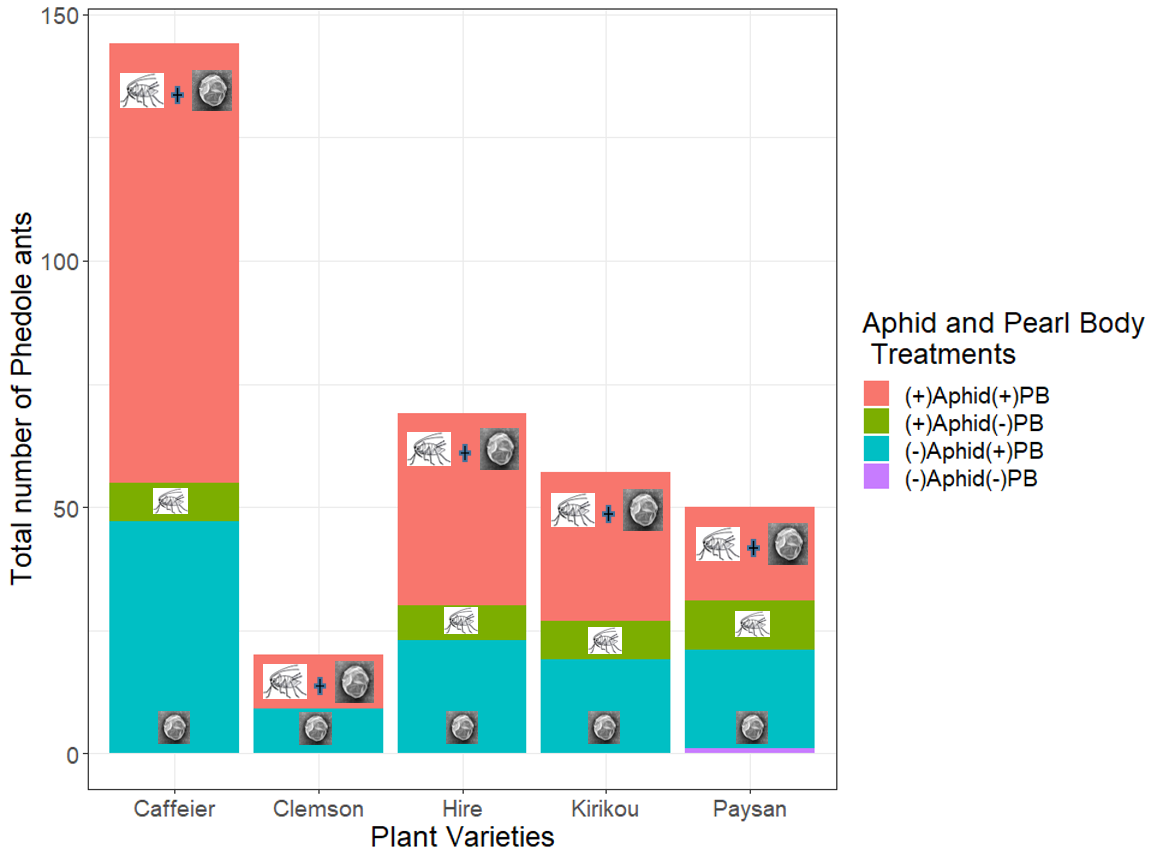


Figure SI 2: Total number of Pheidole ants across different okra varieties in different aphid and pearl body treatment.
